# Dataset on the learning performance of ECDL digital skills of undergraduate students for comparing educational gaming, gamification and social networking^☆^

**DOI:** 10.1016/j.dib.2017.01.017

**Published:** 2017-02-03

**Authors:** Luis de-Marcos, Eva García-López, Antonio García-Cabot

**Affiliations:** Universidad de Alcalá, Dpto. Ciencias de la Computación, Edificio Politécnico, Campus Universitario. Ctra., Barcelona km 33.7. Alcalá de Henares, Madrid, Spain

**Keywords:** Learning, Learning performance, Gamification, Educational game, Social network, ICT education, Digital skills, ECDL

## Abstract

This paper reports data about the learning performance of students using four different motivational tools: an educational game, a gamified plugin, a social networking website and a gamified social networking website. It also reports a control group. The data pertain to 379 students of an undergraduate course that covers basic Information and Communication Technology (ICT) skills in Spain. Data corresponds to different learning modules of the European Computer Driving License (ECDL) initiative. The data include variables of four pre-test scores, four post-test scores and a final examination. It was gathered using a quasi-experimental research design during 2014. Data reported here refers to the research paper in (de-Marcos et al., 2016) [1].

**Specifications Table**

**Table:** 

Subject area	*Education*
More specific subject area	*Information and Communication Technology (ICT) Skills*
Type of data	*Excel*
How data was acquired	*Learning performance indicators of students from assignments and tests*
Data format	*Raw*
Experimental factors	*Data are from a pre-test post-test quasi-experimental design*
Experimental features	*Data includes four pre-test scores, four post test scores, the final examination score, gender and age of participants*
Data source location	*Madrid, Spain*
Data accessibility	*Data is within this article*

**Value of the data**

• Data could be further statistically processed and analyzed.
• Learning indicators reported are based on the ECDL de facto standards for digital skills. Data can be compared with other studies using the same or similar indicators facilitating cross-cultural, age and gender-based analyses.
• Further experimental tools can be designed and tested, using this dataset as a benchmark to compare its effectiveness in terms of learning performance.

## 1 Data

The data file spreadsheet accompanying this article contains 379 rows of data and 12 columns. Each row represents one individual student. The first column describes the experimental tool used by the student. Next columns include gender and age. The eight columns immediately following include pre-test and post-test results of four learning modules of the course. The final column includes the result of a final examination. The first sheet of the data file presents metadata for all variables.

## 2 Experimental design, materials and methods

Data were collected from 379 undergraduate students at the University of Alcalá (Spain). Three experimental tools (treatments) were delivered to students using a quasi-experimental design. A control group using a traditional blended-learning approach was also included. The Ribbonhero educational game (www.ribbonhero.com/) was administered to a group of 75 students. A gamification plugin was administered to a group of 77 students. A social networking website was administered to a group of 75 students. A social gamification web platform was administered to a group of 76 students. The control group had 76 students.

The experimental setting was the undergraduate course on “Qualification for ICT Users” that covers basic computational concepts and digital skills. Syllabus was based on the ECDL/ICDL^1^ certification, which is becoming an international de facto standard for digital skills. Learning performance indicators gathered match four learning modules of the ECDL certification, which are word processor, spreadsheets, presentations and databases. Experimentation took place during Spring 2014 and Autumn 2014. The control group and the experimental groups using gamification and social networking took place during Spring 2014 from February till May. Students of the experimental groups using the educational game and social gamification took the course during Autumn 2014 from October till December. All instances of the course run for 10 weeks from the start without breaks. On the first week of the course, students took a multiple-choice computer pre-test. Experimental conditions were then deployed (weeks 2–9). After completing each learning unit (weeks 3, 5, 7 and 9), students had to submit an individual assignment that measured their learning performance. Assignments measured mostly practical skills and competences related to the use of the computer and the different software tools. Final examination was a written test with 15 questions including multiple choice, fill-in-the-blank and short answer questions. The final examination was designed to assess conceptual learning and it was delivered in week 10. Pre-test results were taken using a computerized test. Lecturers assessed assignments and the final examination using a numeric assessment scale. All marks were normalized to a 0–100 scale. The same conditions and evaluation criteria were used in all groups.

Each learning unit included a document describing concepts and skills, a self-assessment questionnaire, and a set of learning activities. Each learning activity contained a document describing the activity, the digital resources and assets needed to complete it, and sample solutions or visual descriptions of the expected results. Supplementary materials (e.g. videos) and communication tools were also available in the learning platform. All instances of the course were delivered using a blended learning approach. For each of the learning modules students had two lectures in which basic concepts were presented and activities were introduced. After that, students had to work on their own to complete the activities and the assignments using the experimental tool. The gamification plugin and the social gamification web platform provided gamified versions of the learning activities of each learning unit of the course. At the social networking site, lecturers initiated and facilitated discussion about the learning contents and activities. Students were asked to cooperate to complete the activities and to review and discuss activities completed by other students. Learning activities then drove the discussion in the social networking site. The educational game was just offered as Supplementary material that is directly related to the course contents offering additional activities in which students could engage. In the control group, activities were delivered using a traditional learning management system.

The main features, learning approach and targeted benefits of the different instruments were as follows. The educational game was not aligned with the learning objectives of the course. It was intended to promote independent work and exploration from students. The gamification plugin fostered competition [2]. It intended to motivate participation through comparison with peers. The social networking website facilitated cooperation and communication among participants [3]. It was intended to boost participation, collaborative work and community building promoting student-driven discussion. The social gamification web platform was designed to promote both cooperation and competition [1], [4]. It delivered the main features of the gamification plugin and social networking site. Furthermore, social interaction afforded additional means to motivate participation and engagement addressing the needs of different students (modeled as player types [5], [6]) and widening participation.

The main objective guiding experimental design and data collection is to compare the different experimental conditions in terms of learning performance. Existing research presents only limited evidence of the effectiveness of separate approaches. Furthermore, no experiments or studies provide comparable data about educational games, competition- and cooperation-based gamification. Data can be used to analyze the alleged benefits of games-based learning and gamification in terms of how motivation and participation influence learning performance. Detailed experimental design, results and discussion are presented in the research paper to which this data paper refers [1].

## Footnotes

1 ECDL refers to the European Computer Driving License. ICDL is the International Computer Driving License. www.ecdl.org
