# Supplementary material for: Dataset on the learning performance of ECDL digital skills of undergraduate students for comparing educational gaming, gamification and social networking
Source: Data Brief. 2017 Feb 3;11:155–8. doi: 10.1016/j.dib.2017.01.017 (PMC5310199; doi:10.1016/j.dib.2017.01.017)
Supplement: Supplementary file 1 — Supplementary material [file mmc1.pdf]

Manuscript: Dataset on the learning performance of ECDL digital skills of undergraduate students for comparing educational gaming, gamification and social networking

Authors: Luis de-Marcos, Eva García-López, Antonio García-Cabot

The authors whose names are listed immediately below certify that they have NO affiliations with or involvement in any organization or entity with any financial interest (such as honoraria; educational grants; participation in speakers' bureaus; membership, employment, consultancies, stock ownership, or other equity interest; and expert testimony or patent-licensing arrangements), or non-financial interest (such as personal or professional relationships, affiliations, knowledge or beliefs) in the subject matter or materials discussed in this manuscript.

Luis de-Marcos Eva García-López Antonio García-Cabot

29th December 2016
